# Specificity of translocator protein-targeted positron emission tomography in inflammatory joint disease

**DOI:** 10.1186/s13550-020-00736-9

**Published:** 2020-12-07

**Authors:** Yusuf Helo, Graham E. Searle, Federica Borghese, Sonya Abraham, Azeem Saleem

**Affiliations:** 1 Invicro, A Konica Minolta Company, Burlington Danes Building, Hammersmith Hospital, Du Cane Road, London, W12 0NN UK; 2 Imperial College London, Hammersmith Hospital, Du Cane Road, London, W12 0NN UK; 3 Hull York Medical School, Allam Medical Building, University of Hull, Cottingham Road, Hull, HU6 7RX UK

## Abstract

**Objective:**

Expression of the translocator protein (TSPO) on inflammatory cells has facilitated imaging of synovitis with TSPO-targeted positron emission tomography (PET). We aimed to quantitatively assess the specificity of the second-generation TSPO PET radioligand, [^11^C]PBR28, and to generate simplified PET protocols in patients with inflammatory joint disease (IJD) in this pilot study.

**Methods:**

Three IJD patients (two rheumatoid arthritis and one osteoarthritis) with knee involvement underwent dynamic [^11^C]PBR28-PET scans before and after administration of 90 mg of oral emapunil (XBD-173), a TSPO ligand the same day. Radial arterial blood sampling was performed throughout the scan, and total radioactivity and radioactive metabolites were obtained. A semi-automated method was used to generate regions of interest. Standardized uptake value (SUV) and SUV ratio corrected for activity in bone and blood between 50 and 70 min (SUVr_50–70_ bone, SUVr_50–70_ blood, respectively) and PET volume of distribution (*V*_T_) of the radioligand were calculated.

**Results:**

A mean [^11^C]PBR28 radioactivity of 378 (range 362–389) MBq was administered. A significant decrease (*p* < 0.05) in *V*_T_, SUVr_50–70_ bone and SUVr_50–70_ blood observed after oral emapunil confirmed the TSPO specificity of [^11^C]PBR28. A decrease in SUV was not observed in the post-block scan.

**Conclusion:**

[^11^C]PBR28 is TSPO-specific radioligand in IJD patients. Simplified PET protocols with static PET acquisition can be used in the management and evaluation of novel therapeutics that target TSPO overexpressing cells.

## Introduction

Synovial envelope of the articular joint is a critical provider of synovial fluid components and articular cartilage nutrients. Synovial inflammation (synovitis) together with progressive degeneration of articular cartilage is key pathological features in a variety of inflammatory joint diseases (IJDs), as diverse as rheumatoid (RA), psoriatic, juvenile and idiopathic arthritis, lupus and gout and a significant contributor of articular cartilage degeneration in osteoarthritis (OA) [1]. Synovial inflammatory infiltrate is composed of aggressive macrophage- and fibroblast-like mesenchymal cells, macrophage-like cells, fibroblast-like synoviocytes and other inflammatory cells [2, 3].

Detection and treatment of subclinical or early inflammatory arthritis is likely to prevent disease progression, permanent joint damage and associated comorbidity. However, subclinical or early disease is often difficult to detect, leading to a delay in diagnosis [4]. Furthermore, the assessment of response to treatment of inflammatory disease is often based on composite disease activity scores, which can be highly subjective and difficult to reproduce consistently. Therefore, an unmet clinical need is to functionally evaluate the target tissue and assess changes in the infiltrate not only for quantitative assessment but also to evaluate targeted therapies.

Positron emission tomography (PET) studies with the 18 KDa translocator protein (TSPO) radioligands have demonstrated the high expression of TSPO (formerly known as the peripheral benzodiazepine receptor; PBR) on activated macrophages at sites of inflammation, and indeed, up-regulation of TSPO has been noted in macrophages of inflamed synovium of animal models of inflammatory arthritis [5]. Clinical TSPO-PET studies in healthy volunteers and subjects with rheumatoid and psoriatic arthritis have reported higher TSPO radioligand uptake in IJD subjects compared to healthy volunteers [6, 7]. However, to date there have been no clinical studies to confirm that the elevated PET signal can be attributed to specific binding to TSPO in IJD subjects. In this manuscript, we report on a cohort of subjects with IJD who underwent dual [^11^C]PBR28-PET scans prior to and after a heterologous oral TSPO blocking agent, emapunil (XBD-173), to assess the specificity of TSPO binding in the knee joints of the subjects scanned.

## Methods

### Regulatory approval and subjects

The study was approved by the West Midlands–Black Country research ethics committee (ref. 17/WM/0082), Integrated Research Application System (ref. 216737) and Administration of Radioactive Substances Advisory Committee (ref. 630/3925/36195) and conducted in accordance with the Declaration of Helsinki. The study was registered with the UK National Institute of Health Research (NIHR) Clinical Research Network (No. MUSC 33816). Three subjects (two subjects with RA and satisfying the American College of Rheumatology criteria with active clinical disease in the knee and one subject with knee OA) were recruited (Table 1). Participants provided written informed consent, with eligibility determined by medical history, physical examination, coagulation screen and blood genotyping for *rs6971*single-nucleotide polymorphism [8]. Subjects with homozygous G allele (high-affinity binders; HABs) and heterozygous G/A allele at position 439 (Ala147Thr) (medium-affinity binders; MABs) were only included in the study as they have higher binding to [^11^C]PBR28 [8].

**Table 1:** Demographics, disease extent, genotype and activity of [^11^C]PBR28 administered for subjects imaged

Subject number (scan)	Age, gender	Disease	Clinical disease severity and site	Genotype	Activity injected (MBq)
1 (Baseline)	57, F	RA	Right (moderate)	MAB	376.7
1 (Post-block)					382.5
2 (Baseline)	56, F	RA	Bilateral (moderate) Left > right	HAB	389.3
2 (Post-block)					361.7
3 (Baseline)	50, F	OA	Mild left	MAB	374.8
3 (Post-block)					380.8

### PET scanning

[^11^C]PBR28 PET scanning was performed at Invicro, London, on a Siemens PET/CT system Biograph 6 TruePoint with TrueV or a Hi-Rez Biograph 6 (Siemens Healthcare, Erlangen, Germany). Prior to the scan, all patients had a radial arterial cannulation for sampling of blood during the scan and a venous cannula for administration of radioactivity. [^11^C]PBR28 was manufactured as described previously [9].

Initially, a low-dose CT scan of the both knee joints was performed for localization and attenuation correction followed by a 90-min dynamic PET scan after intravenous administration of [^11^C]PBR28. Continuous arterial blood sampling was performed for the initial 15 min; additionally, discrete blood samples were performed for the full duration of the scan to obtain total blood and plasma radioactivity and the fraction of parent radiolabelled compound in the blood. A second [^11^C]PBR28 PET-CT scan was performed the same day after oral administration of the TSPO ligand. Emapunil (XBD-173) was given per orally about 2 h before the second scan.

### Data analysis

**Fig. 1:**
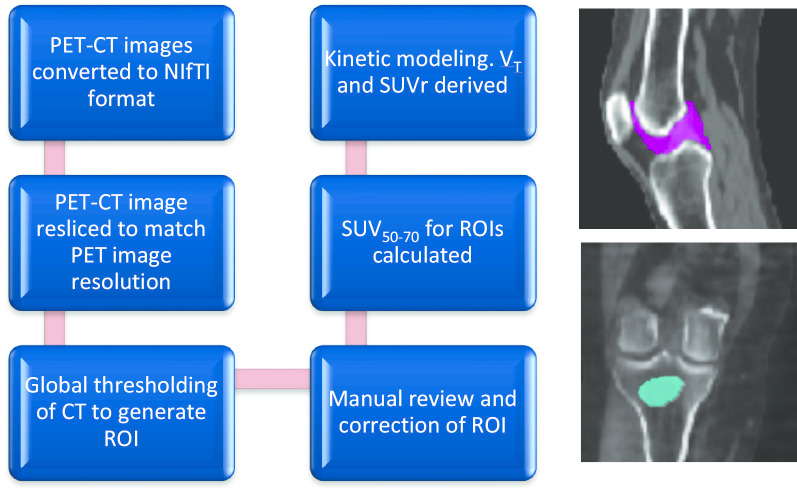
Analysis methodology flowchart illustrating the semi-automated method where a global threshold method was used to define the synovial ROI (sagittal view, pink; top right) and used to calculate uptake parameters. The bone ROI (coronal view, blue; bottom right) is also illustrated

PET images were reconstructed with corrections applied for attenuation, randoms and scatter. All PET-CT images acquired were converted to the NIfTI (Neuroimaging Informatics Technology Initiative) format, and CT images were re-sliced to match the PET image resolution. Synovial regions of interest (ROIs) for each knee were generated using a global threshold method on CT scan, based on the Hounsfield Unit values for bone to exclude bone and adjoining tendon and muscle. For each knee, a single ROI for each knee was generated to include all of the inter-osseous area exclusive of muscle and tendons (Fig. 1). Finally, a manual review of the ROIs was performed by one operator and reviewed by another. Each knee was analyzed separately due to the variation in the severity of joint disease with osteoarthritis and rheumatoid arthritis, as done previously [6].

**Fig. 2:**
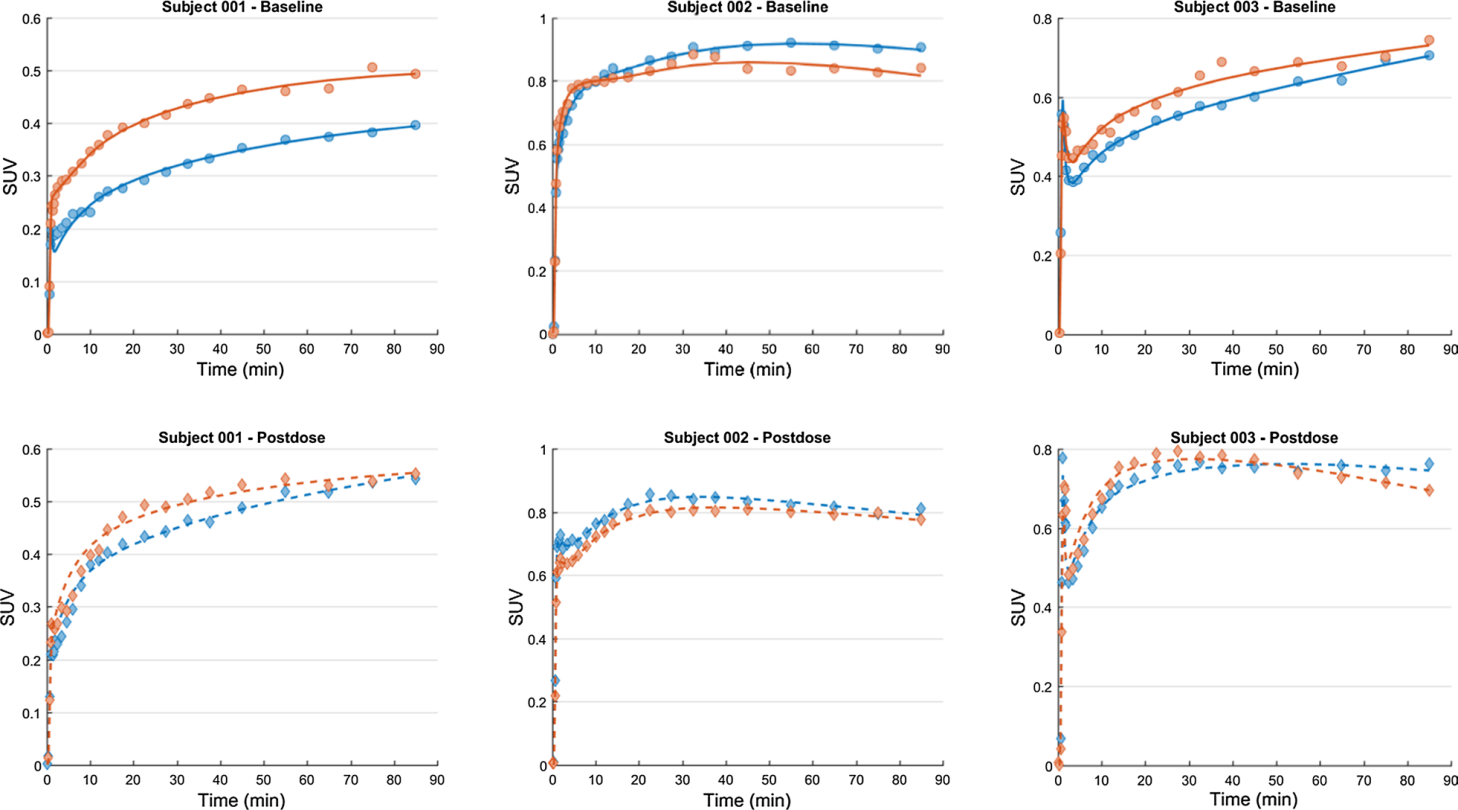
Time–activity plots at baseline (top panel) and post-block (lower panel) depicted as orange (right knee) and blue (left knee) dots and the model fit as continuous lines to the output data using the 2TC model for all the subjects imaged

**Fig. 3:**
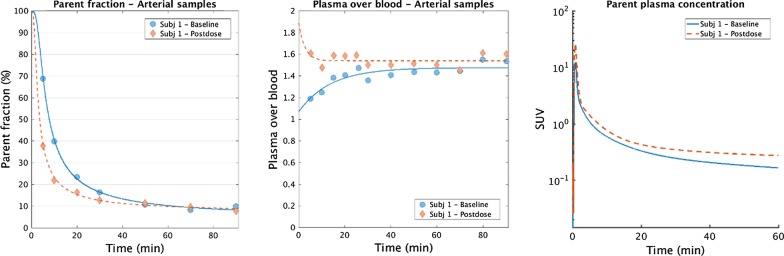
The percentage of parent [^11^C]PBR28 contributing to the total radioactivity (**a**) and the plasma over blood ratio (**b**) in arterial blood that were used to generate the plasma [^11^C]PBR28 input function for the full duration of the scan (**c**) for subject 1 at baseline and post-block

Tissue time–activity curves (TAC) were generated [10] as previously described for each joint synovia (Fig. 2). Radial arterial blood data obtained were used to generate plasma [^11^C]PBR28 TAC for the full duration of the scan (input function) and was modeled with the tissue data (output) (Fig. 3) to generate volume of distribution (*V*_T_) values using a two-tissue compartment model (2TC) as described previously [11, 12].

Semiquantitative uptake values (SUV) were calculated in the synovium between 50 and 70 min (SUV_50–70_) and also normalized to a tissue (bone or blood) uptake between 50 and 70 min (SUVr_50–70_ blood and SUVr_50–70_ bone).

### Statistical analysis

Paired *t *tests were used to compare *V*_T_, SUV or SUVr values within subjects. *p* values less than 0.05 were considered statistically significant.

## Results

### PET analysis

The composite PET image for the full duration of the scan showed radioactivity uptake in the synovia of knee joints, with minimal uptake in the adjoining bone. Review of the TACs (Fig. 2) demonstrated a plateauing in [^11^C]PBR28 SUV between 50- and 70-min post-radioligand injection; hence, SUV and SUVr were calculated between these time points for all subjects. Variability of radioactivity uptake between the two knees post-block was minimal compared to pre-block uptake. *V*_T_ was obtained in all subjects by fitting the 2TC model, apart from one subject (subject 3, Baseline), where the model did not fit the data well.

**Fig. 4:**
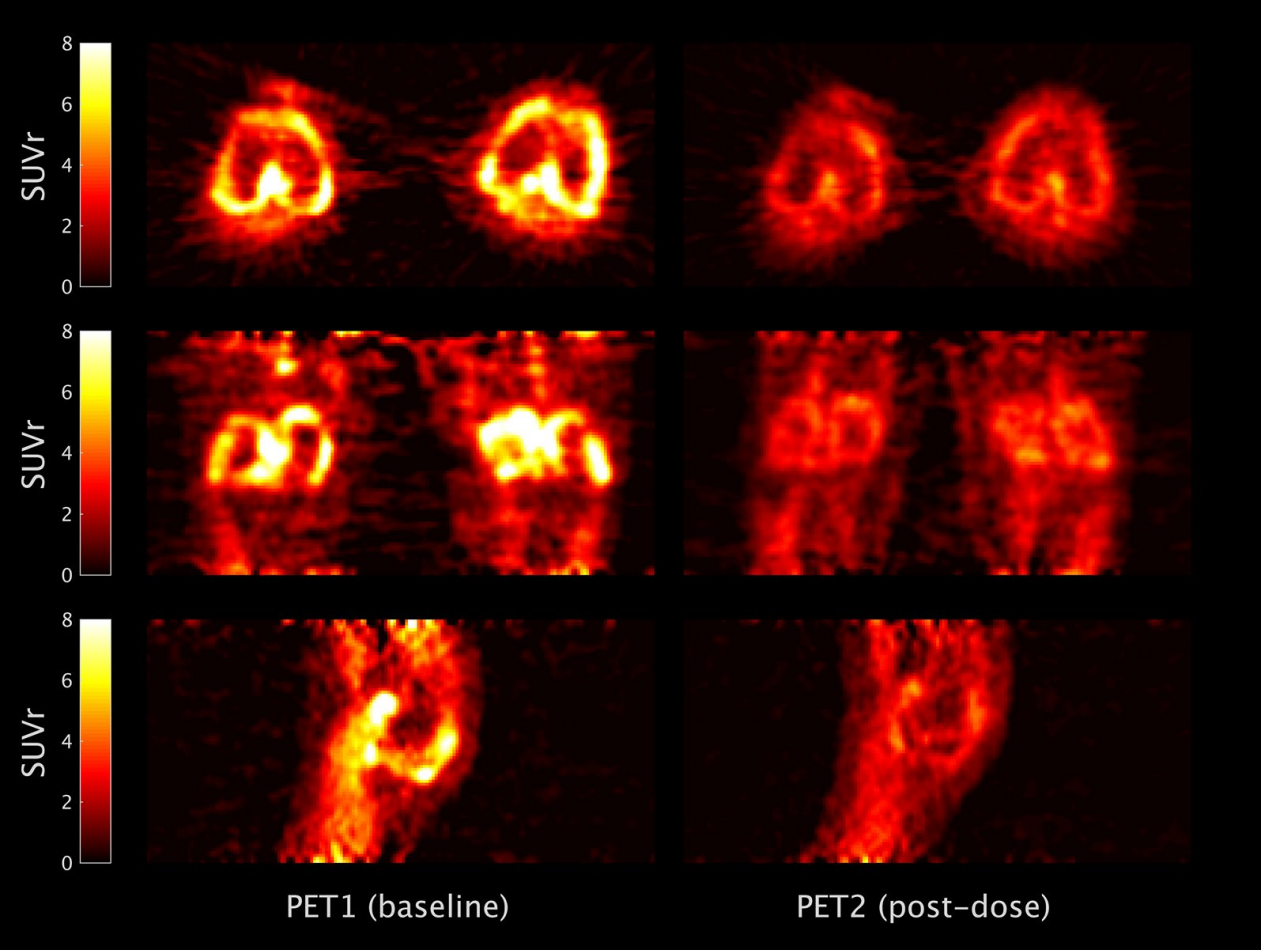
Trans-axial (top), coronal (middle) and sagittal (bottom) images of [^11^C]PBR28 uptake normalized for blood activity (SUVr_50–70_ blood) in subject 2 shows synovial uptake at baseline (PET1 baseline) that is reduced after administration of oral emapunil (PET2 post-block)

There was no change in mean (SD) SUV_50–70_ after oral XBD-173 (0.65 (0.21) versus 0.69 (0.13); *p* = NS). In contrast, PET parameters that account for peripheral distribution of the radioligand such as *V*_T_, SUVr_50–70_ blood and SUVr_50–70_ bone showed a significant decrease in radioligand uptake in the synovium with mean (SD) respective pre- and post-block ligand uptake for *V*_T_ being (4.84 (0.54) versus 2.69 (0.80); *p* < 0.05), for SUVr_50–70_ blood (3.67 (1.29) versus 2.22 (0.32); *p* < 0.05) and for SUVr_50–70_ bone (3.12 (1.03) versus 2.10 (0.20); *p* < 0.05). Individual uptake values for all subjects are provided in Table 2. Figure 4 illustrates a decrease in [^11^C]PBR28 uptake in subject 2 after normalization to blood activity (SUVr_50–70_ blood) after dosing with emapunil.

**Table 2:** Uptake parameters for all the subjects imaged

Subject no. (scan)	SUV		SUVr_50–70_ blood		SUVr_50–70_ bone		*V*_T_	
	Left	Right	Left	Right	Left	Right	Left	Right
1 (Baseline)	0.37	0.46	2.25	2.81	2.14	2.70	5.19	4.32
1 (Post-block)	0.52	0.54	1.90	1.97	1.87	1.91	3.85	2.60
2 (Baseline)	0.92	0.84	5.49	5.01	4.44	4.42	5.40	4.44
2 (Post-block)	0.82	0.80	2.66	2.59	2.36	2.31	2.14	2.18
3 (Baseline)	0.64	0.68	3.13	3.33	2.45	2.60	ND	ND
3 (Post-block)	0.75	0.73	2.13	2.08	2.07	2.10	1.91	1.44

## Discussion

We conducted this study to evaluate if the increased uptake of [^11^C]PBR28 observed in the joints of patients with IJD was specific to TSPO binding and not a consequence of increased radioactivity in joints due to other associated pathological processes. The decrease in the [^11^C]PBR28 quantitative uptake parameter, *V*_T_, after administration of a heterologous TSPO blocking agent confirmed that the increase in [^11^C]PBR28 uptake was specific to TSPO in the tissue cellular infiltrate. However, performing dynamic PET imaging with radial arterial sampling may not always be appropriate as the small joints of the wrist are commonly involved in RA and need to be imaged. Further, performing scans of shorter duration without arterial blood sampling would increase subject tolerability. Since the semiquantitative parameter SUV, which does not account for changes in peripheral metabolism of the radioligand [13], did not exhibit a decrease in uptake after blocking of TSPO, we investigated if we could use a pseudo-reference agent as in the brain where the cerebellum has been used [14]. We used both blood and cancellous bone, both of which contain TSPO [15, 16] as pseudo-reference region as an estimate specific [^11^C]PBR28 tissue uptake. We found that like *V*_T_, a decrease in SUVr_50–70_ blood and SUVr_50–70_ bone was observed after TSPO blocking with XBD-173 was noted with a decrease, indicating that both SUVr_50–70_ blood and SUVr_50–70_ bone could be used to quantify [^11^C]PBR28 uptake. We also confirmed that a static [^11^C]PBR28 scan between 50 and 70 min, correcting for nonspecific binding using bone SUV (SUVr_50–70_ bone) as a pseudo-reference region, allows quantification of TSPO expression with [^11^C]PBR28. This is likely to help facilitate PET studies that provide better subject comfort to evaluate disease and TSPO-targeting therapy and also allow assessment of disease in the small joints of the hand in subjects with IJD.

## Conclusion

In conclusion, we observed that [^11^C]PBR28 behaves as a TSPO-specific radioligand in the knee joint synovia of patients with IJD. PET uptake of [^11^C]PBR28 can also be quantified using simplified acquisition of static PET scans, likely to be suitable in the evaluation of novel therapeutics that target TSPO overexpressing cells in IJD.

## Data Availability

Supporting data will be available on request.

## Abbreviations

HAB: High-affinity binders
IJD: Inflammatory joint disease
OA: Osteoarthritis
MAB: Medium-affinity binders
NIfTI: Neuroimaging informatics technology initiative
NIHR: National Institute of Health Research
PBR: Peripheral benzodiazepine receptor
PET: Positron emission tomography
RA: Rheumatoid arthritis
ROI: Region of interest
SUV: Standardized uptake value
SUVr: Standardized uptake value ratio
TAC: Time–activity curves
TSPO: Translocator protein
*V*_T_: Volume of distribution of a radioligand
2TC: Two-tissue compartment
